# Deep learning-derived retinal biomarker associated with diabetes-related amputation in type 2 diabetes

**DOI:** 10.3389/fendo.2026.1866694

**Published:** 2026-07-01

**Authors:** Junseok Park, Jung Soo Yoon, Sahil Thakur, Dongjin Nam, Sunjin Hwang

**Affiliations:** 1 Seoul National University Hospital, Seoul, Republic of Korea; 2 Mediwhale Inc., Seoul, Republic of Korea; 3 Department of Plastic and Reconstructive Surgery, Myongji Medical Foundation, Myongji Hospital, Ilsan, Republic of Korea; 4 Singapore Eye Research Institute/Singapore National Eye Center, Singapore, Singapore; 5 Department of Internal Medicine, Graduate School, College of Medicine, Yonsei University, Seoul, Republic of Korea; 6 Department of Ophthalmology, Hanyang University Guri Hospital, Guri, Republic of Korea

**Keywords:** artificial intelligence, deep learning, diabetes, retina, amputation, diabetic foot

## Abstract

Diabetic foot (DF)–related amputation remains a major cause of morbidity in patients with type 2 diabetes, yet biomarkers associated with amputation risk remain limited. We aimed to assess the independent association between a deep learning (DL)–derived retinal biomarker using coronary artery calcification (Dr. Noon CVD) and DF–related amputation in patients with type 2 diabetes. This retrospective observational study conducted in a university hospital in South Korea included 392 individuals with type 2 diabetes receiving ophthalmic care (79 with DF-related amputation and 313 without). Participants were randomly split into training (70%) and validation (30%) sets. Model performance in relation to DF-related amputation was assessed using area under the receiver operating characteristic curve (AUC), continuous net reclassification index (cNRI), and integrated discrimination improvement (IDI). Prespecified rule-out (sensitivity ≥0.85) and rule-in (specificity ≥0.90) thresholds were also applied. Adding the retinal biomarker to a basic clinical model showed incremental association with DF-related amputation in the validation set (AUC 0.146, 95% CI: 0.046-0.249; cNRI 0.629, 95% CI: 0.184-1.027; IDI 0.062, 95% CI: 0.012-0.110). The full model achieved an AUC of 0.791, and the association remained consistent across sensitivity analyses using DF risk score derived from the external dataset. Under a 27% amputation prevalence (based on the reported prevalence of amputation among patients with diabetic foot), sensitivity was 87.5% with a negative predictive value of 92.6% at the rule-out cutoff; specificity was 90.4% with a positive predictive value of 56.3% at the rule-in cutoff. A DL-derived retinal biomarker shows meaningful association with DF-related amputation beyond conventional diabetes variables.

## Introduction

Diabetic foot (DF)-related lower-extremity amputation is an extremely disabling and life-threatening complication with an estimated 31% amputation incidence among individuals with DF (1). Five-year mortality after DF-related amputation often exceeds 50%, with cardiovascular disease (CVD) being the most common cause of death (2, 3). Although many amputations are preventable with glycemic control, infection management, and foot-care education, patients often remain unaware of disease progression until the advanced stages (4). Thus, the early identification of high-risk individuals enables timely limb-salvage strategies before irreversible tissue loss and may reduce subsequent mortality.

Recent studies have highlighted the interrelated nature of DF-related amputations and other diabetic vascular complications. For example, microvascular complications such as diabetic retinopathy (DR) have been shown to increase the risk of amputation independent of peripheral artery disease (PAD) (5, 6). Additionally, macrovascular complications such as coronary heart disease may serve as both a predictor of amputation and a condition exacerbated by amputation itself, suggesting a bidirectional relationship (6, 7). These findings suggest that assessing vascular health at accessible sites may help assess the risk of amputation, given the anatomical and physiological parallels among vascular beds (8).

Among the various microvascular beds, the retina offers an accessible and non-invasive window of systemic vascular health. A growing body of research has explored the use of retinal imaging to predict both macro- and microvascular conditions, including CVD, chronic kidney disease (CKD), and even cerebrovascular disorders such as moyamoya disease (9–14). Building on this foundation, Rim et al. developed and validated a deep learning (DL) model that estimated the probability of coronary artery calcium (CAC) from retinal photographs, serving as a proxy for CVD risk (15, 16). The model was trained on over 200,000 paired fundus photographs and CAC measurements and generates a probability-based score (Dr. Noon CVD score) scaled from 0 to 100 using a convolutional neural network architecture. Given the interrelated nature of micro- and macrovascular complications underlying DF-related amputation, this study aimed to evaluate whether a retinal DL-based biomarker captures macrovascular disease burden associated with DF-related amputation in patients with type 2 diabetes.

## Materials and methods

### Main study population (amputation dataset)

We conducted a retrospective observational study including individuals aged 30–79 years who visited the Department of Ophthalmology at a university hospital between 2018 and 2024 and underwent fundus photography, and had a baseline HbA1c measurement within 1 year of fundus imaging (N = 1,481) (**Supplementary Figure 1**). Among these individuals, demographic and clinical variables were identified through electronic medical record review, including age, sex, comorbidities (hypertension, CKD, diabetes mellitus including diabetes type, and prior CVD [defined as myocardial infarction, stroke, or heart failure before the image date]), smoking status, and diabetes duration. Through this process, patients with type 2 diabetes were identified and subsequently classified into two groups according to the occurrence of DF-related amputation between 2016 and 2025: those who underwent DF-related amputation (amputation group; N = 79) and those without (non-amputation group; N = 313). In the amputation group, the fundus photograph was selected if obtained within one year of surgery date and availability of relevant clinical variables. In the non-amputation group, the image was selected if obtained within one year of the availability of clinical measurements. For each image, a DL-derived retinal biomarker for CVD risk was generated using a validated algorithm (15).

### Retinal fundus image acquisition

Bilateral fundus images in the amputation dataset were obtained using a Topcon DRI Optical Coherence Tomography device (DRI OCT-1 Triton^®^, Topcon Corp., Tokyo, Japan), which also provides color fundus photographs. All DL inferences were made using bilateral photographs. DR status was determined using widefield fundus fluorescein angiography (Optos California; Optos plc, Dunfermline, UK) by two retina specialists and classified as “no DR” or “DR” according to Early Treatment Diabetic Retinopathy Study (ETDRS) criteria (17).

### DL-derived retinal biomarker

The retinal biomarker used in this study was derived from the AI-SaMD (Dr. Noon CVD) (15). This DL model was trained using 216,152 paired fundus photographs and CAC scores obtained from Korean health screening centers. The model utilizes a convolutional neural network (CNN) architecture to analyze pixel-wise pathological features within retinal images and to estimate the individual probability of CAC (which is rescaled to a score from 0 to 100) (**Supplementary Figure 2**). The biomarker has been shown to be strongly associated with incident CVD events and validated in three multiethnic external cohorts (15). The predictive performance of this biomarker for CVD events is comparable to that of CAC scoring by computed tomography and superior to carotid ultrasound (16). We verified score scale equivalence across commonly used fundus cameras in internal device-bridging checks (data available upon request). The short-term test-retest repeatability of the biomarker has also been reported previously in the peer-reviewed literature (18). The details of the model training and validation processes are available in previous publications (15, 16).

### Sensitivity analysis using external dataset for DF risk score

**Figure 1:**
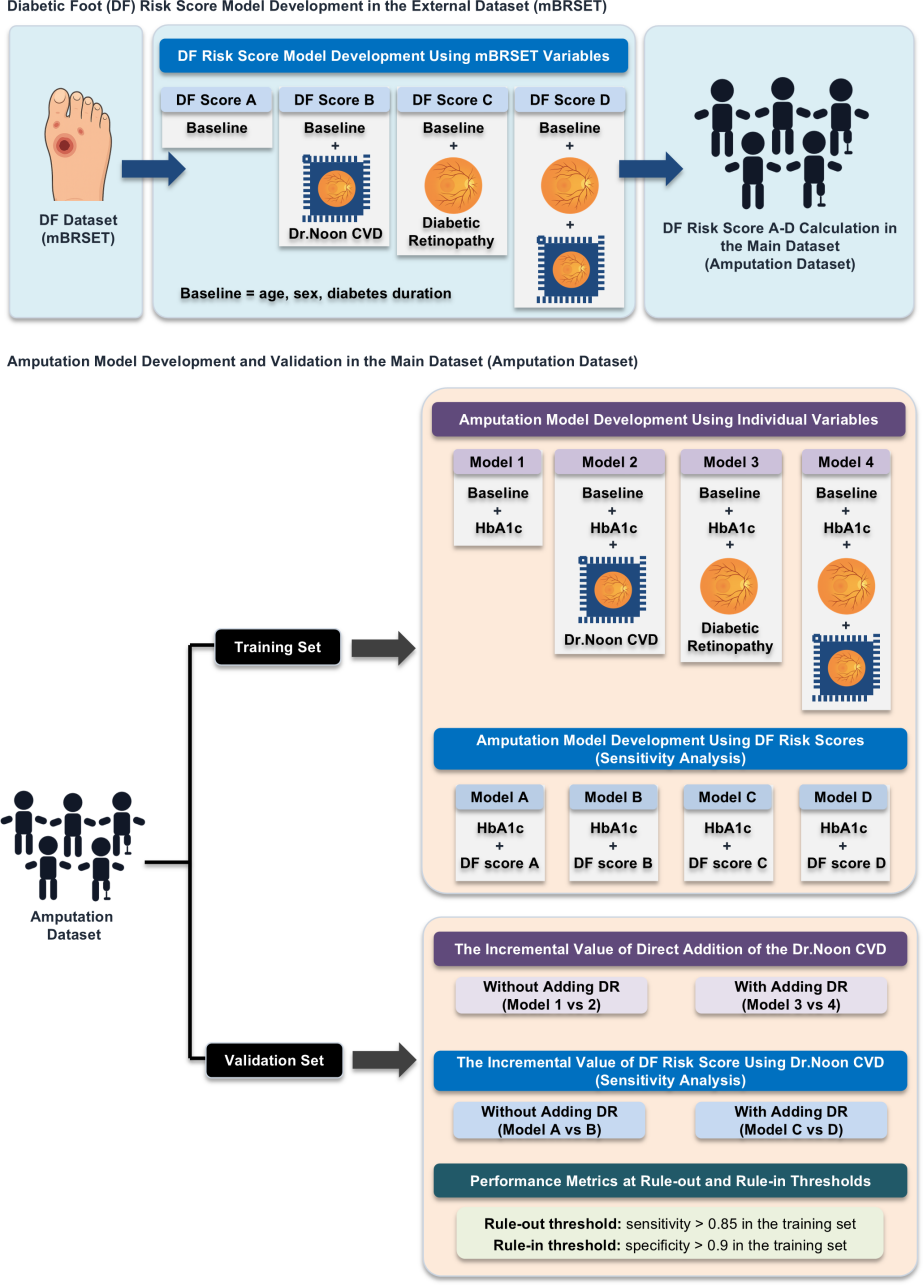
Flowchart of the study design, including model development, internal validation, and external cohort application. CVD, Cardiovascular Disease; DF, Diabetic Foot; DR, Diabetic Retinopathy; HbA1c, Glycated Hemoglobin; mBRSET, Mobile Brazilian Retinal Dataset.

Because amputation represents a downstream consequence of DF, we performed a sensitivity analysis using an external dataset labeled for DF. Using retinal images, we first established a framework to generate a DF risk score from an open-source external dataset (mBRSET; Mobile Brazilian Retinal Dataset (19, 20)). This externally derived DF risk score was then calculated in our original cohort (amputation dataset) and incorporated as an independent variable in the model (**Figure 1**). This approach reflects the biological continuum of diabetes → DF → amputation and mitigates the limited size of our internal dataset. The mBRSET images were obtained using a portable and handheld fundus camera (45°field of view; 1600 × 1600-pixel resolution) after pharmacologic mydriasis^17^. The device showed screening performance comparable to that of standard tabletop cameras for referable DR and macular edema (21).

From the derivation cohort (mBRSET), we included individuals with diabetes based on quality-screened retinal photographs, complete study variables, and no history of CVD (**Supplementary Figure 1**). This external dataset was randomly split into a training partition (80%) and an independent held-out test partition (20%) to develop and internally validate the DF risk models. DF risk scores A–D were constructed using multivariable logistic regression models for the presence of DF. The variable sets were defined as follows: Score A (baseline: age, sex, and diabetes duration), Score B (Baseline + Dr. Noon CVD score), Score C (Baseline + DR), and Score D (Baseline + DR + Dr. Noon CVD score). For each model, the DF risk score corresponded to the model-predicted probability (range 0–1, rescaled to 0–100) of having DF given the included variables; higher values indicated a greater model-implied likelihood of DF.

Model coefficients were estimated in the training partition (80%). The derived models were then applied to the held-out test partition (20%) to evaluate their ability to discriminate DF status using logistic regression performance metrics, thereby confirming that the constructed scores adequately captured DF risk within the mBRSET. For score transfer to the amputation dataset, the DF risk score equations were applied to individuals in the amputation dataset to compute per-person DF risk scores A–D. These scores were then used as independent variables in the amputation models and for comparative evaluation of model performance.

### Study design

#### Study workflow and model specification

The workflow of this study is summarized in **Figure 1**. The amputation dataset was randomly split 70/30 into training and held-out validation sets. In the training set, we developed logistic regression models for amputation risk: models 1 (Baseline + HbA1c), 2 (Baseline + HbA1c + Dr. Noon CVD score), 3 (Baseline + HbA1c + DR), and 4 (Baseline + HbA1c + DR + Dr. Noon CVD score). We also developed models for sensitivity analysis that used pre-derived DF risk scores as independent variables: A (HbA1c + DF risk score A), B (HbA1c + DF risk score B), C (HbA1c + DF risk score C), and D (HbA1c + DF risk score D). Feature importance was summarized from the model coefficients, and all models were evaluated in the held-out validation set for amputation risk.

#### Planned comparisons and analyses

We assessed the discrimination performance of each model and the incremental value of retinal biomarkers by comparing (i) Model 1 vs. 2 (added value of Dr. Noon CVD over the basic model), (ii) Model 3 vs. 4 (added value of Dr. Noon CVD when DR is already included), (iii) Model A vs. B (added value of DF score incorporating the biomarker when DR is excluded), and (iv) Model C vs. D (added value of DF score incorporating the biomarker when DR is included). We also reported the calibration and threshold-based metrics (sensitivity, specificity, positive predictive value (PPV), and negative predictive value (NPV)) for plausible real-world amputation prevalence from prior studies: 1.8% in the general diabetes population (22) and 27% among patients with DF (23). Thresholds were derived in the training set to target a rule-out sensitivity ≥0.85 and a rule-in specificity ≥0.90 and then evaluated in the validation set.

### Statistical analyses

All statistical analyses were performed using R software (version 2024.12.1) with α level of 0.05. Clinical characteristics between the amputation and non-amputation groups, training and validation sets, and DF and non-DF groups were compared using the Wilcoxon rank-sum test for continuous variables and the chi-square test for categorical variables.

In the training set (and the DF derivation dataset), multivariate logistic regression models were developed after checking the variance inflation factors to assess multicollinearity. To evaluate the contribution of retinal biomarkers, we examined the feature importance using standardized regression coefficients from the fitted models. In the validation set, DeLong’s test was used to compare the area under the receiver operating characteristic curve (AUC) across the fitted models. The continuous net reclassification index (cNRI) and integrated discrimination improvement (IDI) were calculated with 95% confidence intervals (Cis) obtained via 2,000 bootstrap resampling.

### Institutional review board

The need for informed consent was waived by the Institutional Review Board (IRB) owing to the retrospective design and use of de-identified patient data. This study adhered to the Declaration of Helsinki and was approved by the IRB of Hanyang University Guri Hospital (IRB No. 2024-12-007-001).

## Results

### Baseline characteristics of the study population

The detailed flowchart of the study is shown in **Figure 1**. A total of 392 individuals with type 2 diabetes were included in the analysis, comprising 79 patients with a history of DF-related amputation and 313 without. The baseline clinical and demographic characteristics are presented in **Table 1**. The amputation group had higher HbA1c levels (median 8.0% (64mmol/mol) vs. 7.1% (54mmol/mol), P < 0.001) and a longer duration of diabetes (> 10 years, 73.4% vs. 52.1%, P = 0.003); these diabetes-related variables were further included as covariates in the subsequent models. The amputation group also showed a greater prevalence of DR (92.4% vs 56.6%, P < 0.001), the Dr. Noon CVD scores were higher compared to non-amputation group (median 50.0 vs 45.0; P < 0.001), and DF risk scores A–D showed the same pattern (medians 17.7–23.3 → 21.4–29.7; all P ≤ 0.004). Consistent with these distributions of risk factors and biomarkers, incident CVD occurred more often in the amputation group and increased with higher retinal CVD scores (**Supplementary Figure 3**, **Supplementary Table 1**). Baseline characteristics across the training and validation sets in the amputation dataset and DF vs non-DF groups in are shown in **Supplementary Tables 2**, **3**. The verification that the DF risk score captured the DF status in the mBRSET is provided in **Supplementary Table 4**.

**Table 1:** Baseline characteristics of participants according to amputation status in the amputation dataset.

Characteristics	Amputation – (N = 313)	Amputation + (N = 79)	P value
Age, median (IQR), Year	60.0 (50.0, 68.0)	58.0 (50.5, 65.0)	0.443
Sex, N (%)			0.579
Male	197 (62.9)	53 (67.1)	
Female	116 (37.1)	26 (32.9)	
Smoking, N (%)			0.674
No	232 (74.1)	61 (77.2)	
Yes	81 (25.9)	18 (22.8)	
Hypertension, N (%)			0.811
No	104 (32.9)	28 (35.4)	
Yes	209 (66.8)	51 (64.6)	
Chronic Kidney Disease, N (%)			1.000
No	180 (57.5)	46 (58.2)	
Yes	133 (42.5)	33 (41.8)	
HbA1c, Median (IQR), % [mmol/mol]	7.1 (6.4, 8.3) [54 (46, 67)]	8.0 (6.9, 10.2) [64 (52, 88)]	< 0.001
Diabetes Duration, Median (IQR)			0.003
0–5 years	96 (30.7)	14 (17.7)	
5–10 years	54 (17.3)	7 (8.9)	
> 10 years	163 (52.1)	58 (73.4)	
Diabetic Retinopathy, N (%)			< 0.001
No	136 (43.5)	6 (7.6)	
Yes	177 (56.6)	73 (92.4)	
Dr. Noon CVD Score, Median (IQR)	45.0 (38.0, 52.0)	50.0 (45.5, 55.0)	< 0.001
DF Risk Score A, Median (IQR)	17.7 (13.1, 23.4)	21.4 (14.4, 26.0)	0.004
DF Risk Score B, Median (IQR)	23.3 (16.5, 29.7)	29.2 (21.7, 34.5)	< 0.001
DF Risk Score C, Median (IQR)	19.2 (14.1, 26.2)	26.2 (19.2, 30.9)	< 0.001
DF Risk Score D, Median (IQR)	22.7 (16.8, 30.1)	29.7 (22.9, 35.8)	< 0.001

CVD, Cardiovascular Disease; DF, Diabetic Foot; DR, Diabetic Retinopathy; HbA1c, Glycated Hemoglobin; IQR, Interquartile Range; N, Number of Individuals.

Wilcoxon and chi-square tests were used to compare differences in the median and distribution of the variables, respectively. The DF risk score was derived from logistic regression models trained on an external dataset and then applied to individuals in the amputation dataset. Four different models were developed to generate the corresponding DF risk scores, each incorporating a different set of variables: scores A (baseline; age, sex, and diabetes duration), B (baseline + Dr. Noon CVD), C (Baseline + DR), and D (baseline + DR + Dr. Noon CVD).

### Feature importance from the fitted models in the training set

**Figure 2:**
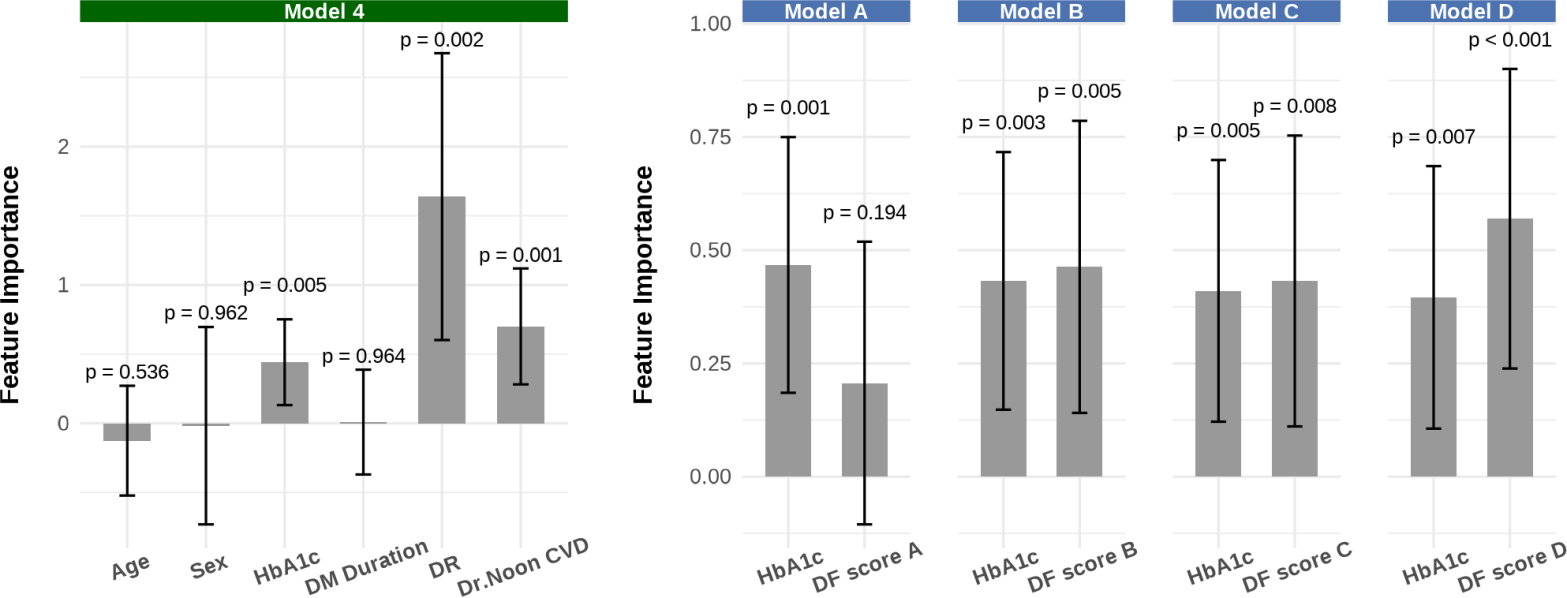
Feature importance of variables for amputation risk in logistic regression models in the training set. AUC, Area Under Curve; CI, Confidence Interval; CVD, Cardiovascular Disease; DF, Diabetic Foot; DM, Diabetes Mellitus; DR, Diabetic Retinopathy; HbA1c, Glycated Hemoglobin. Model 4 included age, sex, HbA1c level, diabetes duration, DR, and Dr. Noon CVD score. Models A-D included HbA1c and DF risk scores A-D. The DF risk score was derived from logistic regression models trained on an external dataset using different sets of variables: Model A (baseline: age, sex, and DM duration), Model B (baseline + Dr. Noon CVD), Model C (Baseline + DR), and Model D (baseline + DR + Dr. Noon CVD). All continuous variables were standardized prior to analysis to allow the comparison of effect sizes. Feature importance was defined as the value of the standardized regression coefficient (β) from the logistic regression model, with error bars representing the 95% confidence intervals.

**Figure 2** presents the standardized logistic regression coefficients for feature importance (regression results are shown in **Supplementary Table 5**). Model 4 (Full model) indicated that DR (β = 1.64, P = 0.002), Dr. Noon CVD (β = 0.70, P = 0.001), and HbA1c (β = 0.44, P = 0.005) were the independent and informative variables; age, sex, and diabetes duration contributed minimally and were not significant. In the DF-proxy models (A–D), DF score A, derived from age, sex, and diabetes duration only, was not informative (β = 0.24, P = 0.194). When DR and/or Dr. Noon CVD were incorporated into the DF score (B–D), the feature importance became significant (β = 0.41, P = 0.003; β = 0.45, P = 0.005; β = 0.57, P < 0.001, respectively). Consistent with this pattern, Dr. Noon CVD showed a marginal association with DF in the mBRSET dataset (aOR: 1.02 per point, 95% CI: 1.00–1.05; P = 0.054; **Supplementary Table 6**).

### Comparison of discriminative performance of the models in the validation set

In the held-out validation set, the addition of the Dr. Noon CVD consistently improved discrimination and reclassification (**Table 2**). Compared with the basic model (Model 1), Model 2 (Model 1 + Dr. Noon CVD) increased AUC by ΔAUC = 0.146 (95% CI, 0.046–0.249), with a positive cNRI of 0.629 (0.184–1.027) and an IDI of 0.062 (0.012–0.110). When DR was already included (Model 3 → 4), Dr. Noon CVD still yielded incremental gains (ΔAUC = 0.081 [0.012–0.150], cNRI = 0.608 [0.173–1.005], IDI = 0.046 [0.003–0.090]). A similar pattern was observed for the DF-proxy models in the sensitivity analysis: incorporating Dr. Noon CVD into the DF score enhanced performance both when DR was excluded (Model A → B: ΔAUC = 0.077 [0.024–0.130], cNRI = 0.759 [0.307–1.165], IDI = 0.038 [0.017–0.061]) and when DR was included (Model C → D: ΔAUC = 0.045 [0.015–0.076], cNRI = 0.676 [0.247–1.092], IDI = 0.026 [0.011–0.041]). The receiver operating characteristic (ROC) curves are shown in **Supplementary Figure 4**.

**Table 2:** Comparison of model discrimination and reclassification improvement in the validation set.

	AUC	ΔAUC (95% CI)	Delong’s *p*	cNRI (95% CI)	IDI (95% CI)
The incremental value of direct addition of the Dr.Noon CVD					
Model 1	0.606	Reference			
Model 2	0.754	0.146 (0.046 – 0.249)	0.004	0.629 (0.184 – 1.027)	0.062 (0.012 – 0.110)
Model 3	0.710	Reference			
Model 4	0.791	0.081 (0.012 – 0.150)	0.021	0.608 (0.173 – 1.005)	0.046 (0.003 – 0.090)
The incremental value of DF risk score using the Dr.Noon CVD					
Model A	0.620	Reference			
Model B	0.697	0.077 (0.024 – 0.130)	0.004	0.759 (0.307 – 1.165)	0.038 (0.017 – 0.061)
Model C	0.684	Reference			
Model D	0.729	0.045 (0.015 – 0.076)	0.004	0.676 (0.247 – 1.092)	0.026 (0.011 – 0.041)

AUC, Area Under Curve; CI, Confidence Interval; cNRI, continuous Net Reclassification Index; CVD, Cardiovascular Disease; DF, Diabetic Foot; DM, Diabetes Mellitus; DR, Diabetic Retinopathy; HbA1c, Glycated Hemoglobin; IDI, Integrated Discrimination Improvement.

The DF risk score was derived from logistic regression models trained on an external dataset and then applied to individuals in the amputation dataset. Four different models were developed to generate the corresponding DF risk scores, each incorporating a different set of variables: scores A (baseline; age, sex, and DM duration), B (baseline + Dr. Noon CVD), C (Baseline + DR), and D (baseline + DR + Dr. Noon CVD). We evaluated incremental values in two ways: (i) the direct addition of Dr. Noon’s CVD to baseline + HbA1c models, without DR (Model 1 vs. 2) and with DR already included (Model 3 vs. 4), and (ii) the increment from using DF risk scores that incorporate Dr. Noon’s CVD—without DR (Model A vs. B) and with DR included (Model C vs. D).

### Prevalence-adjusted risk stratification and threshold performance

**Supplementary Figure 5** shows a non-linear increase in calibrated amputation risk with increasing biomarker levels, supporting the possibility of risk stratification. In **Table 3**, using training-set thresholds targeting rule-out sensitivity ≥0.85 and rule-in specificity ≥0.90, test-set events were 3/57 (5.3%) in *Low*, 13/44 (29.5%) in *Intermediate*, and 8/17 (47.1%) in *High*. At the rule-out cutoff, the sensitivity was 87.5% (specificity, 57.4%), whereas at the rule-in cutoff, the specificity was 90.4% (sensitivity, 33.3%). Assuming a 1.8% population prevalence (amputation prevalence among the general diabetes population), NPV at the rule-out cutoff was 99.60%, and PPV at the rule-in cutoff was 6.00%. Under a higher 27% prevalence (amputation prevalence among individuals with DF), PPV at the rule-in cutoff increased to 56.3%, with NPV at the rule-out cutoff of 92.6%.

**Table 3:** Risk stratification and operating performance in the validation set.

	Low (Rule-out)	Intermediate	High (Rule-in)
Event/N (%)	3/57 (5.3)	13/44 (29.5)	8/17 (47.1)
Sensitivity	87.5%		33.3%
Specificity	57.4%		90.4%
Prior p = 1.8% (Prevalence among individuals with diabetes)			
Posterior p (Mean)	≤0.28% (0.09%)	0.28 – 0.91% (0.53%)	≥0.91% (1.57%)
PPV	3.63%		6.00%
NPV	99.60%		98.67%
Prior p = 27% (Prevalence among individuals with DF)			
Posterior p (Mean)	≤5.27% (1.04%)	5.27 – 15.62% (8.08%)	≥15.62% (23.27%)
PPV	43.20%		56.29%
NPV	92.55%		78.57%

CI, confidence interval; N, Number of individuals; NPV, negative predictive value; PPV, positive predictive value; RR, Relative Risk.

Model 4 was used for the Bayesian calibrated analyses. Prior probabilities were assigned under population prevalence in prior studies to reflect deployment in various prevalence settings (prevalence of 1.8% among individuals with diabetes and 27% among individuals with DF). Tier cut-offs were defined on the training set: rule-out threshold of posterior probability targeting sensitivity ≥ 0.85 (0.28% for prevalence = 1.8%; 5.27% for prevalence = 27%) and rule-in threshold of posterior probability targeting specificity ≥ 0.90 (0.91% for prevalence = 1.8%; 15.62% for prevalence = 27%). These training-derived cutoffs were then applied to the independent validation set to classify the validation set participants into Low, Intermediate, and High tiers. When individuals below the rule-out cutoff were considered non-diseased, the NPV was 99.6% at a prevalence of 1.8% and 92.6% at a prevalence of 27%. Conversely, when individuals above the rule-in cutoff were considered diseased, the PPV was 6.00% at a prevalence of 1.8% and 56.3% at a prevalence of 27%.

## Discussion

In this study, we found that a DL-based retinal biomarker to assess CVD risk using CAC as a training reference also served as an independent factor associated with DF-related amputation. We showed that this biomarker has an association with the risk of amputation in settings where the DR status is unknown using only basic diabetes-related variables such as age, sex, HbA1c, and diabetes duration. Notably, the biomarker showed incremental value even after adding DR status to the model, indicating that it captured systemic vascular risk signals beyond retinal microvascular complications. Its ability to improve risk discrimination was confirmed through internal validation and a sensitivity analysis using an external DF dataset.

In type 2 diabetes, macrovascular complications, such as coronary artery disease, stroke, and PAD, are closely associated with microvascular injury. Chronic hyperglycemia promotes the accumulation of advanced glycation end products and oxidative stress–induced endothelial dysfunction, whereas elevated inflammatory mediators such as interleukin-6 in diabetes trigger chronic vascular wall inflammation, affecting both the micro- and microvasculature (24). In a large Korean population-based study, DR was associated with a 4.03-fold increased risk of amputation, which is a stronger effect than that observed in patients with nephropathy (6). Furthermore, the addition of DR to the discriminative models for PAD–related amputation improved concordance index and NRI values (25). Consistent with this finding, our study also identified DR as a strong marker of amputation risk. These findings support the concept that the retinal microvasculature serves as a “window” into systemic macrovascular burden and its adverse outcomes.

Notably, the DL-derived retinal biomarker remained an independent factor associated with amputation even after adjusting for DR status, underscoring that it captured additional systemic vascular risk signals in the retina. Its association with the amputation risk can be explained by several mechanisms. First, CAC, which is used in the development of the retinal biomarker, can be a marker of amputation risk. Although amputation is more directly related to peripheral arterial calcification, studies have reported that higher CAC scores are significantly associated with lower-limb arterial calcification and amputation, likely because of shared risk factors and common mechanisms in the vascular calcification process (26–31). For example, a previous study reported that amputees had markedly elevated mean CACS (1285 ± 18) compared with control groups (540 ± 84 and 481 ± 11) (28).

An alternative explanation is that the biomarker may capture the continuous cascade from DF to major amputation, which is driven by the complex interplay between microvascular and macrovascular pathology (5, 32). Given that amputation represents the final morbidity of PAD, a type of CVD, the Dr. Noon CVD score may reflect the progressive vascular burden through the following process: hyperglycemia-induced microvascular damage leads to neuropathy and endothelial dysfunction, further increasing the risk of DF, whereas PAD exacerbates ischemia and delays wound healing (33, 34). In line with this, we demonstrated that the biomarker was marginally associated with DF risk after adjusting for vascular disease and that the DF risk scores derived from this biomarker associated with the risk of amputation. Furthermore, the risk of CVD increases during the peri- or post-amputation period as the final stage of this cascade, and biomarkers may capture this increased vascular burden (3, 28, 35). We demonstrated that the biomarker is marginally associated with elevated CVD risk in amputees, which is also supported by reports that cardiovascular status influences and is influenced by amputation with hemodynamic alterations (7, 36, 37).

Our findings indicate that a DL-derived retinal biomarker has the potential to be used across the amputation continuum, from the DF stage through post-amputation follow-up, although further prospective validation is still needed to help strengthen its potential clinical applicability. First, in DF clinics (high prior risk), the three-tier thresholds calibrated in this study provide potential triage: a *Low* tier offers high rule-out value (NPV ≈ 92.6% at DF prevalence ~27%), potentially identifying patients suitable for routine surveillance, whereas a *High* tier was associated with a higher rule-in PPV (≈ 56.3%), which may help identify patients who could benefit from further vascular evaluation and intensified foot care (ankle-brachial index, toe pressures, Duplex ultrasound, wound care, and referral to revascularization). Second, because the biomarker adds information independent of DR, it remains useful when the DR status is unknown or when ophthalmic evaluation is not immediately available (e.g., primary care or community screening). Third, in post-amputation care, higher retinal CVD scores tracked more incident CVD events, suggesting that this biomarker may help identify patients who could benefit from close cardiovascular evaluation and management such as intensive pharmacotherapy. Importantly, this tool may not be intended for the general diabetic population, where a very low prevalence of amputation yields a low PPV; its value is the greatest risk stratification aid in high-amputation-risk populations (e.g., DF).

This study had several limitations. First, due to the retrospective observational and case-control-like study design, the present findings primarily demonstrate an independent association rather than prospective predictive capability. Because patients were classified according to the occurrence of amputation rather than prospectively followed for future events, the reported discrimination performance may not fully reflect real-world clinical performance. Therefore, prospective multicenter external validation studies are required before the biomarker can be considered clinically actionable. Second, although we incorporated an external cohort to mitigate the modest sample size and evaluate the robustness of the findings, the external dataset included DF outcomes rather than independent amputation outcomes and differed from the amputation cohort in several important aspects, including ethnicity, fundus camera type, and the absence of information on diabetes type and glycemic control (HbA1c). Therefore, the external cohort was primarily used as a sensitivity analysis, and larger prospective studies using fully independent datasets with amputation outcomes are required to further validate and generalize these findings. Third, the amputation dataset did not include etiologic variables, such as neuropathy or PAD, body mass index, and lipid profiles. While we showed that retinal biomarkers were marginally associated with DF after adjusting for vascular disease in the external DF dataset, further studies integrating these variables would provide deeper insights.

In conclusion, we demonstrated that a noninvasive DL-based retinal biomarker was independently associated with DF-related amputation in patients with type 2 diabetes. These findings suggest the potential utility of retinal imaging for vascular risk stratification in patients with diabetes, particularly among individuals with a high baseline risk of amputation. Our results also support the broader concept that retinal imaging may provide clinically relevant systemic vascular information beyond ocular disease, although further prospective validation is needed to strengthen its clinical applicability.

## Data Availability

The raw data supporting the conclusions of this article will be made available by the authors, without undue reservation.
