# Supplementary material for: Deep learning-derived retinal biomarker associated with diabetes-related amputation in type 2 diabetes
Source: Front Endocrinol (Lausanne). 2026 Jul 1;17:1866694. doi: 10.3389/fendo.2026.1866694 (PMC13368666; doi:10.3389/fendo.2026.1866694)
Supplement: Supplementary file 1 [file DataSheet1.docx]

**Supplementary Figure 1.** Study flowchart for development and validation of amputation risk models incorporating the retinal biomarker.


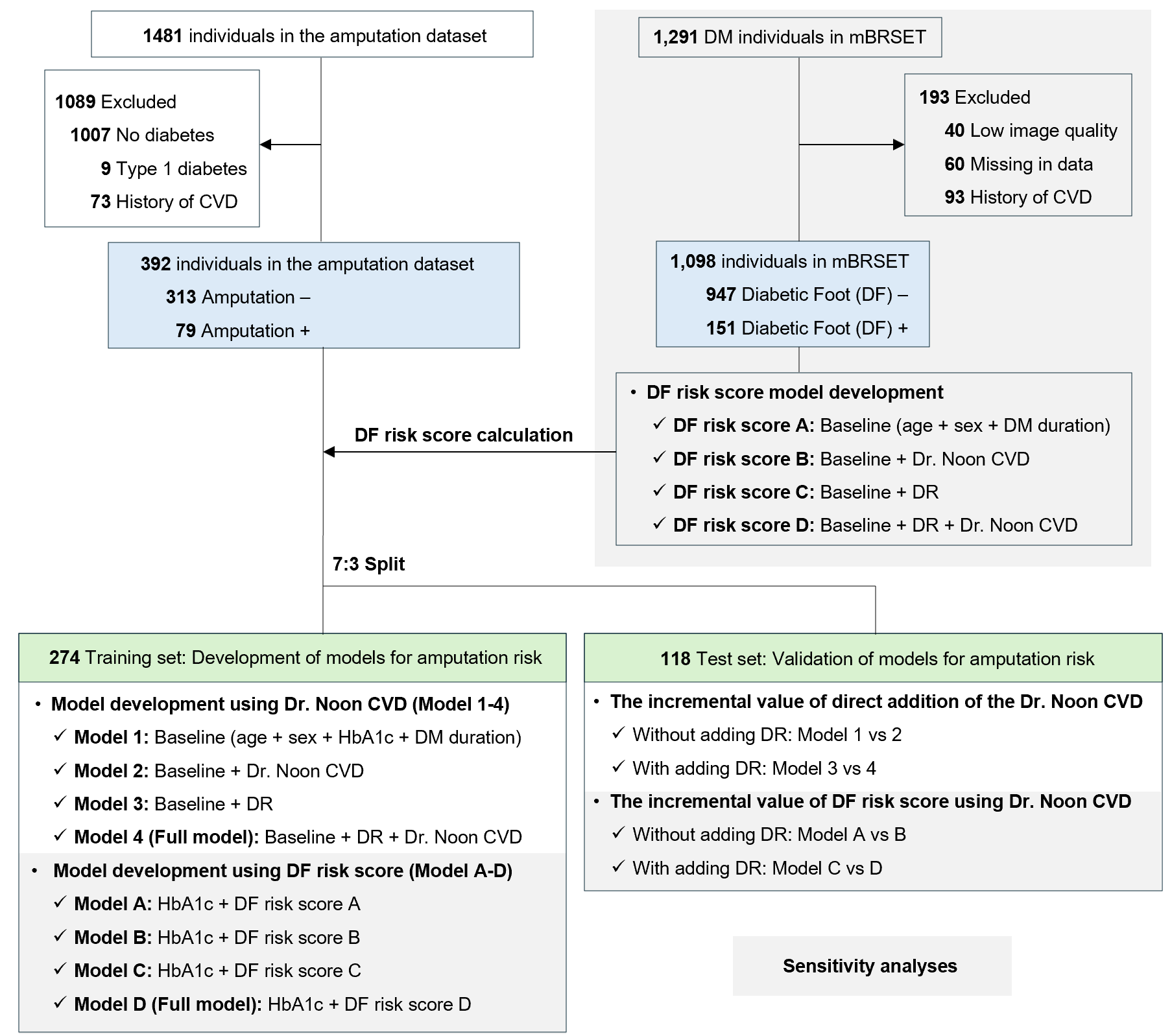


**Abbreviations:** CVD, Cardiovascular Disease; DF, Diabetic Foot; DM, Diabetes Mellitus; DR, Diabetic Retinopathy; HbA1c, Glycated Hemoglobin; mBRSET, Mobile Brazilian Retinal Dataset

The gray-shaded panels represent sensitivity analyses conducted using the external mBRSET dataset, where diabetic-foot risk score models were developed and evaluated.

**Supplementary Figure 2.** AI-Based Retinal Image Analysis: Deep-learning Pipeline for Dr. Noon CVD.

**
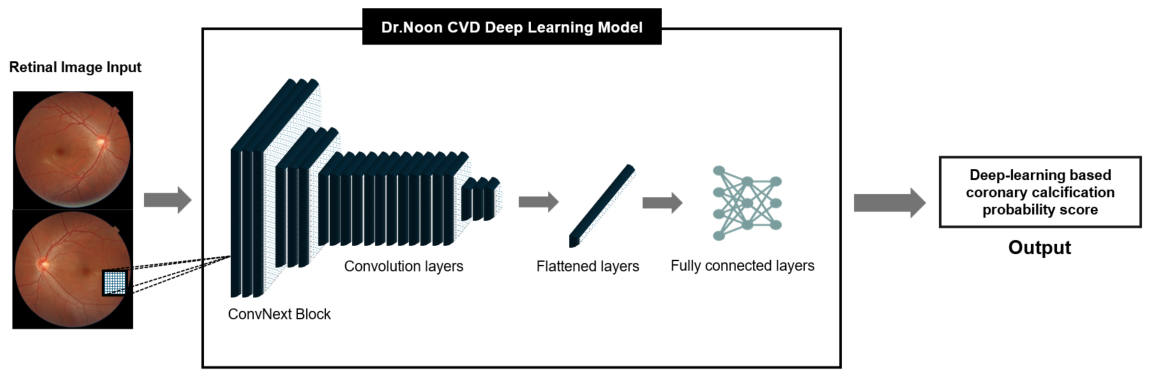
**

**Abbreviations:** CVD, Cardiovascular Disease.

This figure illustrates the architecture of Dr. Noon CVD model, which is a deep learning system designed to estimate cardiovascular risk from retinal images. The process begins with the input of the retinal image into a convolutional neural network, which extracts key visual features. These features are then processed through a series of DL layers, ending in a fully connected layer that generates a probability score indicative of coronary artery calcification. This score has been used in published studies to stratify an individual's CVD risk over both 5-year and 10-year periods.

**Supplementary Figure 3.** Comparison of Dr. Noon CVD scores and the number of incident CVD events between the amputation- and amputation+ groups.


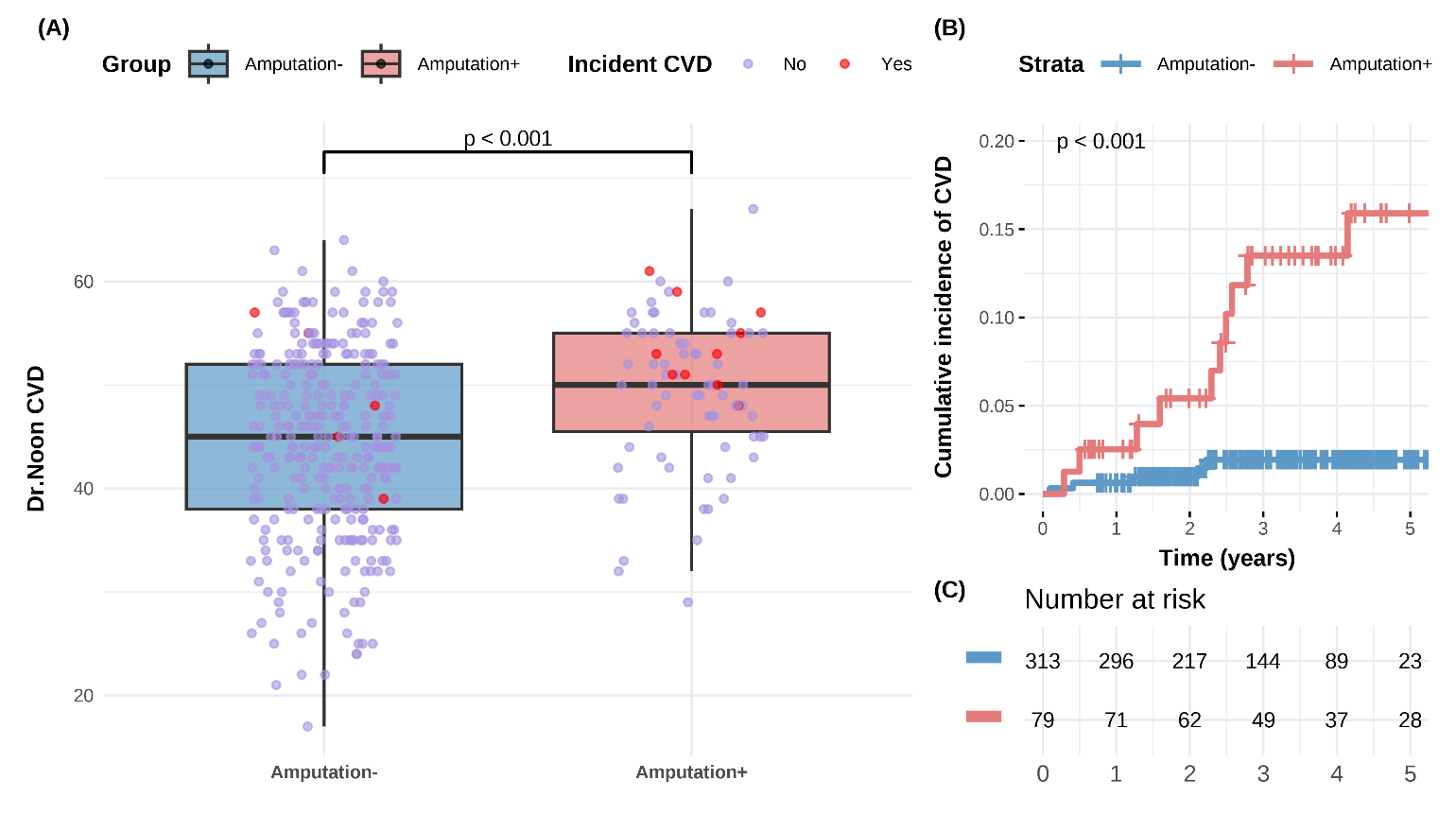


**Abbreviations:** CVD, Cardiovascular Disease.

**(A)** Individual retinal biomarker scores for participants in the amputation and non-amputation groups, with those who experienced subsequent CVD events after the index date indicated in red. The p-value (p < 0.001) obtained from the Wilcoxon test indicated a statistically significant difference between the two groups. Participants who developed incident CVD tended to have higher retinal CVD risk scores regardless of amputation. **(B)** In the amputation group, 5 of 313 participants (1.6%) experienced incident CVD during follow-up (median: 2.97 years), whereas in the amputation+ group, 10 of 79 participants (12.7%) developed CVD.

**Supplementary Table 1.** Cox proportional hazards analysis for incident CVD in the amputation dataset.

|  | **N** | **Events** | **Person-years** | **Crude HR**  **(95% CI)** | ***P* value** | **Adjusted HR**  **(95% CI)** | ***P* value** |
| --- | --- | --- | --- | --- | --- | --- | --- |
| **Age (Year)** | 392 | 15 | 1,268 | 1.03 (0.98, 1.08) | 0.253 | 1.02 (0.97, 1.08) | 0.436 |
| **Sex** |  |  |  |  |  |  |  |
| Male | 250 | 9 | 806 | Reference | - | Reference | - |
| Female | 142 | 6 | 462 | 1.18 (0.42, 3.31) | 0.755 | 1.56 (0.54, 4.54) | 0.416 |
| **Dr. Noon CVD Score (Point)** | 392 | 15 | 1,268 | 1.11 (1.03, 1.19) | 0.007 | 1.08 (0.99, 1.17) | 0.087 |
| **Amputation** |  |  |  |  |  |  |  |
| No | 313 | 5 | 928 | Reference | - | Reference | - |
| Yes | 79 | 10 | 340 | 7.04 (2.40, 20.65) | <0.001 | 5.44 (1.75, 16.91) | 0.003 |

**Abbreviations:** Abbreviations: CI, Confidence Interval; CVD, Cardiovascular Disease; N, Number of participants; HR, Hazard Ratio.

For the non-amputation group, the index date was the retinal imaging date; for the amputation group, it was later retinal imaging and surgery dates. In multivariable Cox models, amputation was strongly associated with incident CVD (aHR, 5.44; 95% CI, 1.75–16.91; P=0.003), whereas the Dr. Noon CVD score showed a borderline per-point association (aHR, 1.08; 95% CI, 0.99–1.17; P=0.087).

**Supplementary Table 2.** Baseline clinical and demographic characteristics in training and validation set.

| **Characteristics** | **Training Set**  **(N = 274)** | **Validation Set**  **(N = 118)** | ***p value*** |
| --- | --- | --- | --- |
| **Age, median (IQR), Year** | 60.0 (51.0, 67.0) | 57.5 (50.0, 68.0) | 0.482 |
| **Sex, N (%)** |  |  | 0.045 |
| Male | 184 (67.2) | 66 (55.9) |  |
| Female | 90 (32.8) | 52 (44.1) |  |
| **Smoking, N (%)** |  |  | 0.939 |
| No | 204 (74.5) | 89 (75.4) |  |
| Yes | 70 (25.5) | 29 (24.6) |  |
| **Hypertension, N (%)** |  |  | 0.774 |
| No | 94 (34.3) | 38 (32.2) |  |
| Yes | 180 (65.7) | 80 (67.8) |  |
| **Chronic Kidney Disease, N (%)** |  |  | 0.573 |
| No | 161 (58.8) | 65 (55.1) |  |
| Yes | 113 (41.2) | 53 (44.9) |  |
| **HbA1c, Median (IQR), % [mmol/mol]** | 7.3 (6.4, 8.5)  [56 (46, 69)] | 7.3 (6.4, 8.6)  [56 (46, 71)] | 0.889 |
| **Diabetes duration, Median (IQR)** |  |  | 0.343 |
| 0–5 years | 73 (26.6) | 37 (31.4) |  |
| 5–10 years | 47 (17.2) | 14 (11.9) |  |
| > 10 years | 154 (56.2) | 67 (56.8) |  |
| **Diabetic Retinopathy, N (%)** |  |  | 0.863 |
| No | 98 (35.8) | 44 (37.3) |  |
| Yes | 176 (64.2) | 74 (62.7) |  |
| **Amputation, N (%)** |  |  | 1.000 |
| No | 219 (79.9) | 94 (79.7) |  |
| Yes | 55 (20.1) | 24 (20.3) |  |
| **Dr. Noon CVD Score, Median (IQR)** | 46.0 (40.0, 52.0) | 46.0 (39.0, 52.0) | 0.580 |
| **DF Risk Score A, Median (IQR)** | 19.1 (13.9, 24.4) | 17.5 (12.6, 23.7) | 0.191 |
| **DF Risk Score B, Median (IQR)** | 25.4 (18.2, 30.6) | 23.7 (16.8, 29.9) | 0.140 |
| **DF Risk Score C, Median (IQR)** | 20.8 (15.5, 28.1) | 20.0 (13.6, 27.5) | 0.273 |
| **DF Risk Score D, Median (IQR)** | 24.3 (17.8, 31.8) | 23.8 (16.0, 30.9) | 0.253 |

**Abbreviations:** CVD, Cardiovascular Disease; DF, Diabetic Foot; HbA1c, Glycated Hemoglobin; IQR, Interquartile Range; N, Number of Individuals.

The dataset was split into training and validation sets (7:3 ratio) using 51 seeds. Wilcoxon and chi-square tests were used to compare differences in the median and distribution of the variables, respectively. The DF risk score was derived from logistic regression models trained on an external dataset and then applied to individuals in the amputation dataset. Four different models were developed to generate the corresponding DF risk scores, each incorporating a different set of variables: scores A (baseline; age, sex, and DM duration), B (baseline + Dr. Noon CVD), C (Baseline + DR), and D (baseline + DR + Dr. Noon CVD).

**Supplementary Table 3.** Baseline clinical and demographic characteristics in mBRSET.

| **Characteristics** | **Diabetic Foot –**  **(N = 947)** | **Diabetic Foot +**  **(N = 151)** | ***p value*** |
| --- | --- | --- | --- |
| **Age, median (IQR), Year** | 63.0 (55.0, 69.0) | 60.0 (53.0, 67.0) | 0.480 |
| **Sex, N (%)** |  |  | 0.001 |
| Male | 309 (32.6) | 70 (46.4) |  |
| Female | 638 (67.4) | 81 (53.6) |  |
| **Smoking, N (%)** |  |  | 0.964 |
| No | 886 (93.6) | 142 (94.0) |  |
| Yes | 61 (6.4) | 9 (6.0) |  |
| **Hypertension, N (%)** |  |  | 0.940 |
| No | 282 (29.8) | 46 (30.5) |  |
| Yes | 665 (70.2) | 105 (69.5) |  |
| **Chronic Kidney Disease, N (%)** |  |  | 0.042 |
| No | 923 (97.5) | 142 (94.0) |  |
| Yes | 24 (2.5) | 9 (6.0) |  |
| **Diabetes duration, Median (IQR)** |  |  | 0.004 |
| 0–5 years | 434 (45.8) | 49 (32.5) |  |
| 5–10 years | 236 (24.9) | 41 (27.2) |  |
| > 10 years | 277 (29.3) | 61 (40.4) |  |
| **Diabetic Retinopathy, N (%)** |  |  | < 0.001 |
| No | 762 (80.5) | 100 (66.2) |  |
| Yes | 185 (19.5) | 51 (33.8) |  |
| **Vascular Disease, N (%)** |  |  | 0.001 |
| No | 819 (86.5) | 115 (76.2) |  |
| Yes | 128 (13.5) | 36 (23.8) |  |
| **Dr. Noon CVD Score, Median (IQR)** | 29.3 (25.8, 34.1) | 32.0 (25.6, 36.9) | 0.014 |
| **DF Risk Score A, Median (IQR)** | 12.6 (9.2, 16.0) | 14.8 (11.1, 20.7) | < 0.001 |
| **DF Risk Score B, Median (IQR)** | 12.2 (8.8, 15.8) | 14.9 (11.1, 21.1) | < 0.001 |
| **DF Risk Score C, Median (IQR)** | 11.8 (9.0, 16.3) | 15.1 (10.5, 21.5) | < 0.001 |
| **DF Risk Score D, Median (IQR)** | 11.7 (8.8, 15.8) | 14.7 (10.3, 213) | < 0.001 |

**Abbreviations:** CVD, Cardiovascular Disease; DF, Diabetic Foot; HbA1c, Glycated Hemoglobin; IQR, Interquartile Range; mBRSET, Mobile Brazilian Retinal Dataset; N, Number of Individuals.

Wilcoxon and chi-square tests were used to compare differences in the median and distribution of the variables, respectively. The DF risk score was derived from logistic regression models trained on an external dataset (mBRSET) and then applied to individuals in the amputation dataset. Four different models were developed to generate the corresponding DF risk scores, each incorporating a different set of variables: scores A (baseline; age, sex, and DM duration), B (baseline + Dr. Noon CVD), C (Baseline + DR), and D (baseline + DR + Dr. Noon CVD).

**Supplementary Table 4.** Results of logistic regression models for diabetic foot in the test set of mBRSET**.**

| **Covariates** | **Crude OR**  **(95% CI)** | ***P* value** |
| --- | --- | --- |
| **DF Risk Score A (Point)** | 1.11 (1.04 – 1.19) | 0.002 |
| **DF Risk Score B (Point)** | 1.12 (1.05 – 1.20) | < 0.001 |
| **DF Risk Score C (Point)** | 1.11 (1.04 – 1.17) | < 0.001 |
| **DF Risk Score D (Point)** | 1.11 (1.04 – 1.17) | < 0.001 |
|  |  |  |

**Abbreviations:** CI, Confidence Interval; DF, Diabetic Foot; mBRSET, Mobile Brazilian Retinal Dataset; OR, Odds Ratio.

To verify that the DF risk score captured the DF status in the mBRSET, we divided the mBRSET into a training set (80%) and a test set (20%). After fitting the DF risk score models in the training set, we applied them to the individuals in the validation set and conducted logistic regression analysis. Four different models were developed to generate the corresponding DF risk scores, each incorporating a different set ofvariables: scores A (baseline; age, sex, and DM duration), B (baseline + Dr. Noon CVD), C (Baseline + DR), and D (baseline + DR + Dr. Noon CVD).

**Supplementary Table 5.** Adjusted odds ratios for amputation risk according to clinical variables in the training set.

| **Covariates** | **Adjusted OR**  **(95% CI)** | ***P* value** |
| --- | --- | --- |
| **Model 4** |  |  |
| **Age (Year)** | 0.99 (0.96 – 1.02) | 0.541 |
| **Sex** |  |  |
| Male | Reference | - |
| Female | 0.99 (0.47 – 2.01) | 0.972 |
| **HbA1c (%)** | 1.26 (1.07 – 1.50) | 0.005 |
| **Diabetes Duration** |  |  |
| 0–5 years | Reference | - |
| 5–10 years | 0.44 (0.11 – 1.53) | 0.219 |
| > 10 years | 0.90 (0.38 – 2.22) | 0.821 |
| **Diabetic Retinopathy** |  |  |
| No | Reference | - |
| Yes | 5.16 (1.96 – 16.52) | 0.002 |
| **Dr. Noon CVD Score (Point)** | 1.08 (1.03 – 1.14) | 0.001 |
| **Model A** |  |  |
| **HbA1c (%)** | 1.28 (1.10 – 1.50) | 0.001 |
| **DF Risk Score A (Point)** | 1.03 (0.98 – 1.08) | 0.194 |
| **Model B** |  |  |
| **HbA1c (%)** | 1.26 (1.08 – 1.47) | 0.003 |
| **DF Risk Score B (Point)** | 1.06 (1.02 – 1.10) | 0.005 |
| **Model C** |  |  |
| **HbA1c (%)** | 1.24 (1.07 – 1.45) | 0.005 |
| **DF Risk Score C (Point)** | 1.06 (1.01 – 1.10) | 0.008 |
| **Model D** |  |  |
| **HbA1c (%)** | 1.23 (1.06 – 1.44) | 0.007 |
| **DF Risk Score D (Point)** | 1.07 (1.03 – 1.11) | < 0.001 |

**Abbreviations:** CI, Confidence Interval; CVD, Cardiovascular Disease; DF, Diabetic Foot; HbA1c, Glycated Hemoglobin; OR, Odds Ratio.

Model 4 included age, sex, HbA1c level, diabetes duration, DR, and Dr. Noon CVD score. The DF risk score was derived from logistic regression models trained on an external dataset using different sets of variables: A (baseline: age, sex, and diabetes duration), B (baseline + Dr. Noon CVD), C (Baseline + DR), and D (baseline + DR + Dr. Noon CVD). Models A–D are amputation models that include HbA1c and DF risk scores. The adjusted odds ratios and 95% confidence intervals from the multivariate logistic regression model are shown in the table.

**Supplementary Table 6.** Crude and adjusted odds ratios for diabetic foot according to mBRSET risk factors.

| **Covariates** | **Crude OR**  **(95% CI)** | ***P* value** | **Adjusted OR**  **(95% CI)** | ***P* value** |
| --- | --- | --- | --- | --- |
| **Age (Year)** | 0.99 (0.97 – 1.00) | 0.071 | 0.97 (0.96 – 0.99) | 0.001 |
| **Sex** |  |  |  |  |
| Male | Reference | - | Reference | - |
| Female | 0.56 (0.40 – 0.79) | 0.001 | 0.55 (0.38 – 0.80) | 0.002 |
| **Diabetes Duration** |  |  |  |  |
| 0–5 years | Reference | - | Reference | - |
| 5–10 years | 1.54 (0.98 – 2.40) | 0.057 | 1.56 (0.98 – 2.45) | 0.056 |
| > 10 years | 1.95 (1.30 – 2.93) | 0.001 | 1.99 (1.30 – 3.08) | 0.002 |
| **Vascular Disease** |  |  |  |  |
| No | Reference | - | Reference | - |
| Yes | 2.00 (1.31 – 3.02) | 0.001 | 2.20 (1.41 – 3.37) | < 0.001 |
| **Dr. Noon CVD Score (Point)** | 1.03 (1.01 – 1.06) | 0.006 | 1.02 (1.00 – 1.05) | 0.054 |

**Abbreviations:** CI, Confidence Interval; CVD, Cardiovascular Disease; mBRSET, Mobile Brazilian Retinal Dataset; OR, Odds Ratio.

The adjusted odds ratios and 95% confidence intervals from the multivariate logistic regression model are shown in the table.

**Supplementary Figure 4.** Receiver operating characteristic curves of the models for discriminating amputation in the validation set.


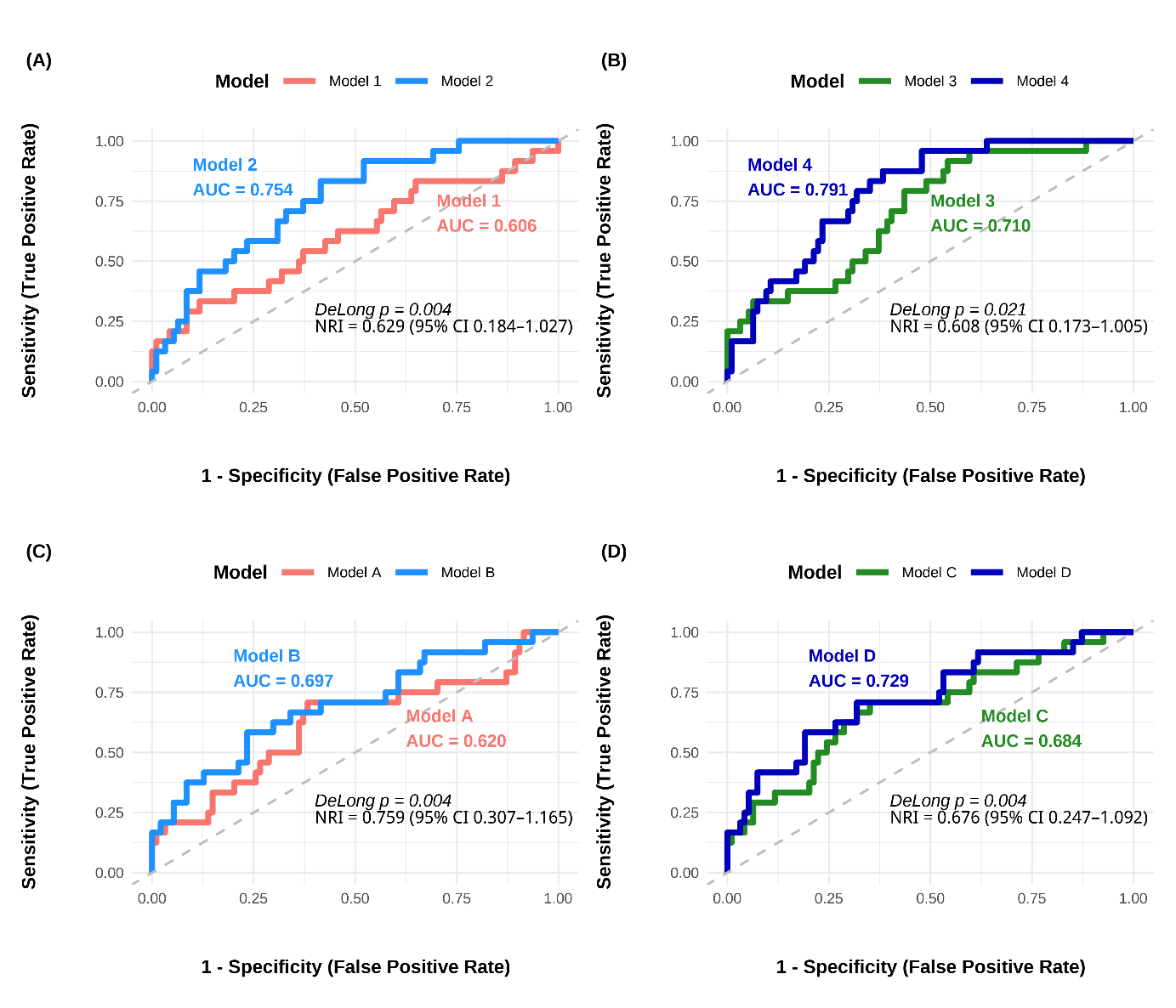


**Abbreviations:** AUC, Area Under Curve; CI, Confidence Interval; DR, Diabetic Retinopathy; NRI, Net Reclassification Index.

Receiver operating characteristic (ROC) curves for logistic regression models discriminating amputation. DF scores A-D represent four different models to generate the corresponding DF risk scores, each incorporating a different set of scores: A (baseline; age, sex, and diabetes duration), B (Baseline + Dr. Noon CVD), C (Baseline + DR), and D (Baseline + DR + Dr. Noon CVD).

- **(A)** Model 1 (Baseline + HbA1c) vs. model 2 (baseline + HbA1c + Dr. Noon CVD): AUC 0.606 vs. 0.754; DeLong *p* = 0.004; continuous NRI = 0.629 (95% CI 0.184–1.027).
- **(B)** Model 3 (Baseline + HbA1c + DR) vs. Model 4 (baseline + HbA1c + DR + Dr. Noon CVD): AUC 0.710 vs. 0.791; DeLong *p* = 0.021; continuous NRI = 0.608 (95% CI 0.173–1.105).
- **(C)** Model A (HbA1c + DF risk score A) vs. Model B (HbA1c + DF risk score B) — AUC 0.620 vs. 0.697; DeLong *p* = 0.004; continuous NRI = 0.759 (95% CI 0.307–1.165).
- **(D):** Model C (HbA1c + DF risk score C) vs. Model D (HbA1c + DF risk score D): AUC, 0.684 vs. 0.729; DeLong *p* = 0.004; continuous NRI, 0.676 (95% CI, 0.247–1.092).

**Supplementary Figure 5.** Bayesian-calibrated partial dependence of amputation risk across Dr. Noon CVD scores on real-world amputation prevalence in different settings.


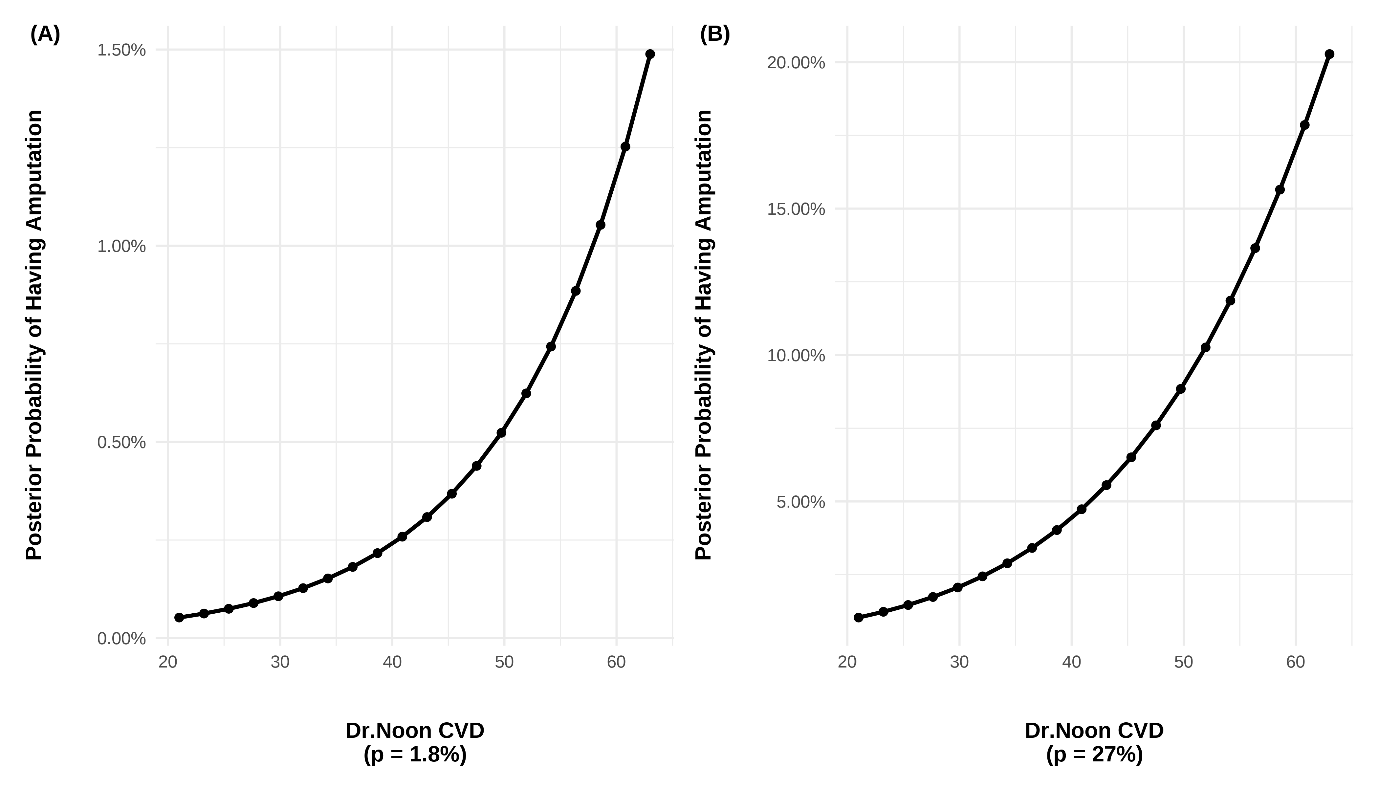


**Abbreviations:** CVD, Cardiovascular Disease.

Partial dependence plot showing the relationship between biomarkers and the posterior probability of amputation after Bayesian recalibration using real-world amputation prevalence (A: 1.8% in individuals with diabetes; B: 27% in individuals with DF). Model 4 was used for the Bayesian calibrated analyses. Each point represents the average predicted probability of amputation when the biomarker is fixed at that value and the other covariates are held constant. The results demonstrated a nonlinear increase in calibrated amputation risk as the biomarker increased, suggesting its potential value for risk stratification.
